# Supplementary material for: The German validation of the Body Perception Questionnaire-Short Form (BPQ-SF) and its relation to current self-report measures of interoception
Source: PLOS Ment Health. 2024 Jun 4;1(1):e0000038. doi: 10.1371/journal.pmen.0000038 (PMC12798462; doi:10.1371/journal.pmen.0000038)
Supplement: S1 Text — (DOCX) [file pmen.0000038.s001.docx]

**Supporting information**

**S1: Item characteristics for the individual items of the BPQ-SF**

**Table A. Descriptives of the German version of the BPQ-SF at the item level (validation sample).**

| Item | Mean | SD | Skewness | Kurtosis | Minimum | Maximum |
| --- | --- | --- | --- | --- | --- | --- |
| BPQ_01 | 2.7 | 1.2 | 0.22 | 1.96 | 1 | 5 |
| BPQ_02 | 3.5 | 1.2 | -0.43 | 2.11 | 1 | 5 |
| BPQ_03 | 3.4 | 1.2 | -0.38 | 2.25 | 1 | 5 |
| BPQ_04 | 3.1 | 1.1 | -0.15 | 2.18 | 1 | 5 |
| BPQ_05 | 3.8 | 1.2 | -0.81 | 2.76 | 1 | 5 |
| BPQ_06 | 3.5 | 1.1 | -0.42 | 2.30 | 1 | 5 |
| BPQ_07 | 2.7 | 1.3 | 0.07 | 1.81 | 1 | 5 |
| BPQ_08 | 3.9 | 1.1 | -0.97 | 3.16 | 1 | 5 |
| BPQ_09 | 3.5 | 1.2 | -0.50 | 2.35 | 1 | 5 |
| BPQ_10 | 2.2 | 1.3 | 0.69 | 2.22 | 1 | 5 |
| BPQ_11 | 2.8 | 1.1 | 0.01 | 2.34 | 1 | 5 |
| BPQ_12 | 3.8 | 1.2 | -0.70 | 2.45 | 1 | 5 |
| BPQ_13 | 4.0 | 1.1 | -0.93 | 3.06 | 1 | 5 |
| BPQ_14 | 3.7 | 1.1 | -0.70 | 2.74 | 1 | 5 |
| BPQ_15 | 3.3 | 1.3 | -0.28 | 1.98 | 1 | 5 |
| BPQ_16 | 2.9 | 1.3 | 0.01 | 1.84 | 1 | 5 |
| BPQ_17 | 2.7 | 1.4 | 0.27 | 1.68 | 1 | 5 |
| BPQ_18 | 3.3 | 1.1 | -0.20 | 2.23 | 1 | 5 |
| BPQ_19 | 2.9 | 1.2 | 0.03 | 1.97 | 1 | 5 |
| BPQ_20 | 2.4 | 1.2 | 0.51 | 2.20 | 1 | 5 |
| BPQ_22 | 3.8 | 1.0 | -0.63 | 2.68 | 1 | 5 |
| BPQ_23 | 2.2 | 1.3 | 0.77 | 2.35 | 1 | 5 |
| BPQ_24 | 3.5 | 1.0 | -0.43 | 2.59 | 1 | 5 |
| BPQ_25 | 2.5 | 1.3 | 0.47 | 2.02 | 1 | 5 |
| BPQ_26 | 3.1 | 1.1 | -0.16 | 2.21 | 1 | 5 |
| BPQ_27 | 2.7 | 1.3 | 0.15 | 1.88 | 1 | 5 |
| BPQ_28 | 1.4 | 0.8 | 1.95 | 6.33 | 1 | 5 |
| BPQ_29 | 1.9 | 1.0 | 0.97 | 3.13 | 1 | 5 |
| BPQ_30 | 1.8 | 0.9 | 0.95 | 3.29 | 1 | 5 |
| BPQ_31 | 1.5 | 0.7 | 1.48 | 5.27 | 1 | 5 |
| BPQ_32 | 2.0 | 1.0 | 0.75 | 3.03 | 1 | 5 |
| BPQ_33 | 1.6 | 0.9 | 1.35 | 4.27 | 1 | 5 |
| BPQ_34 | 1.3 | 0.7 | 2.34 | 8.63 | 1 | 5 |
| BPQ_35 | 1.2 | 0.5 | 3.27 | 15.04 | 1 | 5 |
| BPQ_36 | 1.2 | 0.5 | 3.82 | 19.41 | 1 | 5 |
| BPQ_37 | 1.8 | 0.9 | 1.09 | 4.06 | 1 | 5 |
| BPQ_38 | 1.1 | 0.5 | 4.42 | 26.11 | 1 | 5 |
| BPQ_39 | 1.5 | 0.8 | 1.88 | 6.45 | 1 | 5 |
| BPQ_40 | 1.8 | 0.9 | 1.11 | 3.73 | 1 | 5 |
| BPQ_41 | 1.4 | 0.8 | 1.97 | 6.69 | 1 | 5 |
| BPQ_42 | 1.6 | 0.7 | 1.24 | 4.19 | 1 | 5 |
| BPQ_43 | 1.8 | 1.0 | 1.27 | 4.21 | 1 | 5 |
| BPQ_44 | 1.8 | 0.9 | 1.08 | 4.08 | 1 | 5 |
| BPQ_45 | 1.8 | 1.0 | 1.19 | 4.02 | 1 | 5 |
| BPQ_46 | 1.9 | 1.0 | 1.16 | 3.95 | 1 | 5 |
