# Supplementary material for: The German validation of the Body Perception Questionnaire-Short Form (BPQ-SF) and its relation to current self-report measures of interoception
Source: PLOS Ment Health. 2024 Jun 4;1(1):e0000038. doi: 10.1371/journal.pmen.0000038 (PMC12798462; doi:10.1371/journal.pmen.0000038)
Supplement: S2 Text — (DOCX) [file pmen.0000038.s002.docx]

**S2: German version of the BPQ-SF**

**Table A. Items from Body Awarenes (Bewusstsein) subscale**

| Bitte bewerten Sie, wie bewusst Sie sich folgender Zustände sind. Wählen Sie diejenige Antwort, welche für Sie am ehesten zutrifft.  Während der meisten Situationen bin ich mir der folgenden Dinge bewusst… |
| --- |
| 1. Häufiges Schlucken |
| 1. Der Drang zu husten, um meinen Hals freizubekommen |
| 1. Dass mein Mund trocken ist |
| 1. Wie schnell ich atme |
| 1. Dass meine Augen feucht werden oder tränen |
| 1. Geräusche, die mit meiner Verdauung zu tun haben |
| 1. Ein Anschwellen meines Körpers oder einzelner Körperteile |
| 1. Stuhldrang |
| 1. Muskelverspannungen in Armen und Beinen |
| 1. Ein aufgedunsenes Gefühl durch Wassereinlagerungen |
| 1. Angespannte Gesichtsmuskulatur |
| 1. Gänsehaut |
| 1. Magen- und Bauchschmerzen |
| 1. Völlegefühl oder Aufgeblähtsein |
| 1. Schwitzige Hände |
| 1. Schweiß auf der Stirn |
| 1. Zittern der Lippen |
| 1. Schweiß in den Achselhöhlen |
| 1. Die Temperatur meines Gesichts (besonders der Ohren) |
| 1. Zähneknirschen |
| 1. Allgemeine Nervosität |
| 1. Die Nackenhaare stellen sich auf |
| 1. Konzentrationsschwierigkeiten |
| 1. Schluckzwang |
| 1. Wie stark mein Herz schlägt |
| 1. Verstopfungsgefühl |

**Table B. Items from the Autonomous Nervous System Reactivity (Reaktionsfähigkeit des Autonomen Nervensystems) subscale.**

| Das autonome Nervensystem ist derjenige Teil Ihres Nervensystems, welcher Ihr Herzkreislauf-, Atmungs- und Verdauungssystem sowie Ihre Körpertemperatur reguliert. Es ist ebenfalls am Erleben und am Ausdruck von Gefühlen beteiligt. Das autonome Nervensystem funktioniert individuell unterschiedlich.  Diese Skala wurde entwickelt, um zu messen, wie Ihr autonomes Nervensystem reagiert.  Bitte bewerten Sie, wie häufig die folgenden Aussagen auf Sie zutreffen: |
| --- |
| 1. Ich habe Schwierigkeiten das Atmen mit dem Essen zu koordinieren. |
| 1. Wenn ich esse, habe ich Schwierigkeiten zu sprechen. |
| 1. Mein Herz schlägt oft unregelmässig. |
| 1. Wenn ich esse, fühlt sich das Essen trocken an und klebt in Mund und Hals. |
| 1. Ich fühle mich kurzatmig. |
| 1. Ich habe Schwierigkeiten, das Atmen mit dem Sprechen zu koordinieren. |
| 1. Wenn ich esse, habe ich Schwierigkeiten das Schlucken, Kauen und/oder Saugen mit meiner Atmung zu koordinieren. |
| 1. Ich habe einen andauernden Husten, der mich beim Sprechen und Essen beeinträchtigt. |
| 1. Ich ersticke beinahe am Speichel in meinem Mund. |
| 1. Ich habe Schmerzen in der Brust. |
| 1. Ich ersticke beinahe beim Essen. |
| 1. Wenn ich spreche, kriege ich oft das Gefühl, ich müsse husten oder den Speichel im Mund herunterschlucken. 1 |
| 1. Wenn ich atme, habe ich den Eindruck, nicht genug Sauerstoff zu kriegen. |
| 1. Ich habe Schwierigkeiten, meine Augen zu kontrollieren. |
| 1. Ich fühle mich, als ob ich erbrechen müsste. |
| 1. Ich habe einen „übersäuerten“ Magen. |
| 1. Ich bin verstopft. |
| 1. Ich leide an einer Magenverstimmung (Verdauungsstörung). |
| 1. Nach dem Essen leide ich an Verdauungsproblemen. |
| 1. Ich habe Durchfall. |
