# Supplementary material for: The German validation of the Body Perception Questionnaire-Short Form (BPQ-SF) and its relation to current self-report measures of interoception
Source: PLOS Ment Health. 2024 Jun 4;1(1):e0000038. doi: 10.1371/journal.pmen.0000038 (PMC12798462; doi:10.1371/journal.pmen.0000038)
Supplement: Attachment — Submitted filename: ReviewersComments_280324.docx [file pmen.0000038.s003.docx]

**Reviewer #1**:

**This is an interesting and easy to read study. The authors have collected a unique dataset using a sound methodology. The paper is generally well written and structured.
However,
- I feel this unique dataset has not been utilised or discussed to its full extent.**

We thank the reviewer for the kind words. We would like to mention that the dataset used in the current study is very extensive and includes many different variables. From the current dataset, different research questions have arisen that ended up in different publications (Brand et al., 2023; Tünte et al., 2024).

As mentioned throughout the paper and in the preregistration (https://aspredicted.org/HC1_D39), we exclusively aim at validating the BPQ-(V)SF. For this reason, we decided not to make use of the full dataset, but only considered the variables relevant for our purposes. However, we would also like to mention that the data of the current and previously published studies have been or will be made available in public repositories to explore other related research questions.

- **The role of co-authors may be included.**

We thank the reviewer for pointing this out. We now report the co-authors’ contributions at the end of the manuscript.

*S.B., M.R.T., and C.V.B. contributed to conceptualization, data curation, formal analysis, investigation, methodology, project administration, resources, validation, visualization, writing—original draft, and writing—review, and editing. M.W., S.H., and M.W. contributed to resources, supervision, and writing—review, and editing.*

**Reviewer #2:**

**The paper has been well-written.**

We thank the reviewer for the kind words.

**- The manuscript lacks key words.**

We apologize for not providing key words before. We now added them to the main text.

Key words: *Subjective* *Interoception, BPQ, BPQ-SF, validation, Anxiety, Depression.*

**- All indices must have a specified threshold, especially Table 2 requires captions.**

We apologize for not providing this beforehand. We now marked the indices based on specified thresholds, according to the existing guidelines (Comrey & Lee, 2013) (i.e., indices larger than .6 in bold, indices larger .45 in italics and indexes lower than .45 left unformatted)

**- Selecting a loading option below is not appropriate. What is 60? The goal of this work is to validate a questionnaire in the German language. However, the loadings are inadequate for validating the questionnaire.**
We thank the reviewer for pointing this out. We now mentioned this limitation in the discussion. We agree that some items do not pass the suggested threshold according to current guidelines (e.g., Comrey & Lee, 2013). However, due to the good fit indices we assume that the scale is a reliable tool to measure subjective interoception. Nevertheless, we now argue in the discussion that the scale could be further improved in future research:

*It should be noted that, as for other versions of the BPQ-SF, in the German version not all items loaded optimally to the three factors according to current guidelines [60], suggesting that the questionnaire may benefit from further refinement.*

**- Authors should aim to clarify the cross loadings, Principal Component Analysis (PCA), and Exploratory Factor Analysis (EFA) for questionnaire validation**.
We thank the reviewer for making us aware of this point. We assume that there might be a misunderstanding as we did not perform an EFA but directly performed a CFA. We based our decision on existing guidelines (e.g., Moosbrugger & Kelava, 2012, p. 340), which suggest that “If a theory is available, the CFA and not the EFA should always be used. This is the only way to confirm the hypotheses and assess the quality of the resulting model.” – as the BPQ-SF is a widely used questionnaire with an existing rationale, we aimed to confirm this rationale. Therefore, no cross loadings exist in the used model. To avoid additional confusion, we added the term “Confirmatory Factor Analysis” in the heading of Table 2.

**- The study's implications should also be provided.**

We thank the reviewer for pointing this out. We now provided the potential implications of the study.

*Two main implications can be extracted from our results. First, improving our understanding of the ways in which existing self-report measures of interoception are related can help to clarify the taxonomy of interoception in a more comprehensive and inclusive manner. Second, highlighting the associations between different facets of interoception (e.g., autonomic reactivity) and psychopathological tendencies, may help promote the development intervention programs that tackle specific interoceptive abilities to improve mental health.*

References
Moosbrugger, H. & Kelava, A. (Hrsg.). (2020). *Testtheorie und Fragebogenkonstruktion* (3., vollst. neu bearb., erw. u. akt. Auflage 2020). Springer Berlin; Springer.

Comrey, A.L., & Lee, H.B. (1992). *A First Course in Factor Analysis* (2nd ed.). Psychology Press. https://doi.org/10.4324/9781315827506
